# Reversible thrombocytopenia during hibernation originates from storage and release of platelets in liver sinusoids

**DOI:** 10.1007/s00360-021-01351-3

**Published:** 2021-03-04

**Authors:** Edwin L. de Vrij, Hjalmar R. Bouma, Maaike Goris, Ulrike Weerman, Anne P. de Groot, Jeroen Kuipers, Ben N. G. Giepmans, Robert H. Henning

**Affiliations:** 1 Department of Clinical Pharmacy and Pharmacology, University Medical Center Groningen, University of Groningen, Hanzeplein 1, 9700 RB Groningen, The Netherlands; 2 Department of Plastic Surgery, University Medical Center Groningen, Groningen, the Netherlands; 3 Department of Internal Medicine, University Medical Center Groningen, University of Groningen, Groningen, the Netherlands; 4 Department of Biomedical Sciences of Cells and Systems, University Medical Center Groningen, University of Groningen, Groningen, the Netherlands

**Keywords:** Platelet, Thrombosis, Torpor, Temperature, Liver, Storage

## Abstract

Immobility is a risk factor for thrombosis due to low blood flow, which may result in activation of the coagulation system, recruitment of platelets and clot formation. Nevertheless, hibernating animals—who endure lengthy periods of immobility—do not show signs of thrombosis throughout or after hibernation. One of the adaptations of hemostasis in hibernators consists of a rapidly reversible reduction of the number of circulating platelets during torpor, i.e., the hibernation phase with reduction of metabolic rate, low blood flow and immobility. It is unknown whether these platelet dynamics in hibernating hamsters originate from storage and release, as suggested for ground squirrel, or from breakdown and de novo synthesis. A reduction in detaching forces due to low blood flow can induce reversible adhesion of platelets to the vessel wall, which is called margination. Here, we hypothesized that storage-and-release by margination to the vessel wall induces reversible thrombocytopenia in torpor. Therefore, we transfused labeled platelets in hibernating Syrian hamster (*Mesocricetus auratus*) and platelets were analyzed using flow cytometry and electron microscopy. The half-life of labeled platelets was extended from 20 to 30 h in hibernating animals compared to non-hibernating control hamsters. More than 90% of labeled platelets were cleared from the circulation during torpor, followed by emergence during arousal which supports storage-and-release to govern thrombocytopenia in torpor. Furthermore, the low number of immature platelets, plasma level of interleukin-1α and normal numbers of megakaryocytes in bone marrow make platelet synthesis or megakaryocyte rupture via interleukin-1α unlikely to account for the recovery of platelet counts upon arousal. Finally, using large-scale electron microscopy we revealed platelets to accumulate in liver sinusoids, but not in spleen or lung, during torpor. These results thus demonstrate that platelet dynamics in hibernation are caused by storage and release of platelets, most likely by margination to the vessel wall in liver sinusoids. Translating the molecular mechanisms that govern platelet retention in the liver, may be of major relevance for hemostatic management in (accidental) hypothermia and for the development of novel anti-thrombotic strategies.

**Supplementary Information:**

The online version contains supplementary material available at 10.1007/s00360-021-01351-3.

## Introduction

Immobility in humans bears an increased risk of thrombosis (Engbers et al. 2014)—even in healthy subjects as exemplified by the up to fourfold increased relative risk of deep vein thrombosis (DVT) after 4 h of immobility during travel (Cannegieter et al. 2006). Platelets are crucial in the development of both venous and arterial thrombosis. In humans, increased DVT risk by immobility is due to reduced venous flow, inducing hypoxia and subsequent activation of the endothelium, staging a scaffold for adherence of platelets and coagulation factors firing off the coagulation cascade and inducing thrombus formation (Brill et al. 2013; Montoro-Garcia et al. 2016). Dislodging of such a thrombus upon restoration of blood flow may cause life-threatening pulmonary embolism (White 2003). In addition to venous thromboembolic complications, platelets are involved in arterial thrombus formation during relative stasis of blood, specifically in the cardiac atria in atrial fibrillation (Gosk-Bierska et al. 2016; Watson et al. 2009), which is associated with an increased risk of cerebrovascular accidents with a 30 day mortality as high as 55% (Bekwelem et al. 2015). Furthermore, platelets can initiate thrombosis in vasculitides and in atherosclerotic blood vessels by adhering to the inflamed, activated endothelium (Emmi et al. 2015; Nieswandt et al. 2011).

Curiously, immobility induced thromboembolism is absent in hibernators, in spite of several risk factors being present throughout hibernation. Hibernation is used by many mammalian species to survive extreme environments (Carey et al. 2003). Hibernation is characterized by torpor phases with extreme reduction of metabolism leading to a large decrease in amongst others heart and respiratory rate, as well as in body temperature (Carey et al. 2003). Torpor bouts last several days to weeks and are interspersed by short phases of arousal, wherein metabolism and other physiological parameters fully recover. All hibernators are immobile during the torpor phase and some species even remain immobile during arousal phases until springtime (Carey et al. 2003; Cooper et al. 2016b; Utz et al. 2009). At face value, hibernators would suffer an increased risk of thrombosis because of the presence of several risk factors for thrombosis, including obesity in the pre-hibernation phase (Martin 2008), and immobility (Carey et al. 2003), reduced blood flow (Bullard and Funkhouser 1962) and increased blood viscosity during torpor (Halikas and Bowers 1973; Kirkebo 1968). Hibernation is associated with crucial changes in the hemostatic system during torpor consistent with a reduced risk of thrombosis, amongst others by reducing platelet count with more than 90% and reducing coagulation factors, such as factor VIII and IX, suppressing blood clotting (Cooper et al. 2012; de Vrij et al. 2014; Lechler and Penick 1963). Although hibernation is associated with suppressed hemostasis during torpor, presumably to preclude inadvertent formation of thromboembolisms, the risk of bleeding lurks during arousal if changes are not reversed timely. Therefore, torpid squirrel and hamster for instance rapidly recover platelet count within 2 h of arousal (de Vrij et al. 2014; Lechler and Penick 1963; Pivorun and Sinnamon 1981) and adequately recover (although not completely restore) whole blood clotting tendency, as measured by thromboelastography (Cooper et al. 2012).

We hypothesize that reduction and reversal of circulating platelet count during hibernation is due to storage and release via platelet margination to the vessel wall in well vascularized organs, rather than by breakdown of platelets and de novo synthesis. Main arguments are that platelet count rapidly normalizes within a few hours of arousal, i.e., faster than accounted for by synthesis from megakaryocytes, and that the amount of newly synthesized platelets does not increase in arousal (Cooper et al. 2012; de Vrij et al. 2014). Recently, a histological analysis of hibernating ground squirrels points towards the storage and release of platelets in liver in torpid ground squirrels (Cooper et al. 2017). Whether megakaryocyte rupture, recently discovered as a rapid platelet producing process (Nishimura et al. 2015), might play a role in the swift recovery of platelet count during arousal is not yet known. In this study, we set out to identify platelet storage and release as the mechanism governing platelet dynamics in hibernating hamsters and to disclose the major locations involved. By assessing the number of platelets in circulation and in organs in time by flow cytometry and large scale electron microscopy (EM) in hibernating hamsters transfused with 5-chloromethylfluorescein diacetate (CMFDA)-labeled platelets, we demonstrate that trombocytopenia in torpor is governed by storage and release of platelets, most likely in liver sinusoids. Examination of immature platelet amounts and bone marrow megakaryocytes reveals no signs of de novo synthesis of platelets to account for the rapid and major recovery in platelet count during arousal, while low levels of D-dimer and platelet activation markers reduce the likeliness of thrombus formation during hibernation.

## Materials and methods

### Animals

Syrian hamsters (*Mesocricetus auratus*, age 3 months) were obtained from Envigo USA and individually housed at ‘summer’ photoperiod light:dark cycle (L:D) of 14 h:10 h at 20–22 °C with free access to standard laboratory chow and water until induction of hibernation. Animal work was approved by the Institutional Animal Ethical Committee of the University Medical Center Groningen.

### Hibernation in hamsters

After 7 weeks at ‘summer’ photoperiod, hamsters were housed at ‘autumn’ photoperiod (L:D of 8 h:16 h at 20 °C) for 7 weeks, followed by reduction of ambient temperature to 5 °C and housing under constant darkness (‘winter’ period) (de Vrij et al. 2014). Passive infrared sensors coupled to a computer system monitored individual movements. Hamsters were euthanized at different stages of euthermia or hibernation: summer euthermia (SE), winter euthermia (WE), early torpor (TE), late torpor (TL), early arousal (AE) and late arousal (AL). Summer and winter euthermia were defined as a euthermic body temperature (approximately 37 °C) during 'summer' and ‘winter’ photoperiods in absence of any torpor bouts. Early and late torpor were defined as 24–48 and > 48 h of immobility, respectively, and confirmed in all animals by oral temperature measurements. Early and late arousal were defined as 1.5 h and > 8 h after induced arousal, and a body temperature of ≥ 35 °C.

### Blood samples

Blood was obtained under isoflurane 2% in air/O_2_ anesthesia from the abdominal aorta into one-tenth volume of 3.2% sodium citrate or in lithium heparin coated tubes. Cell count was performed on a Sysmex PoCH 100-iv analyzer. Immature platelet fraction was determined with a Sysmex XE-2100 by staining RNA using the RET-SEARCH (II) dye followed by quantification of reticulated cells (Ko et al. 2013). Plasma was prepared by whole blood centrifugation at 3000*g* × 15 min at 22 °C and stored at -80 °C. D-dimer was measured with a Modular analyzer (Roche Diagnostics) with reagents from Roche.

### Allogeneic labeled platelet transfusion

Donor blood was diluted 1:1 (v/v) in Buffered Saline Glucose Citrate (116 mM NaCL, 13.6 mM Na_2_Citrate, 8.6 mM Na_2_HPO_4_, 1.6 mMKH_2_PO_4_, 11.1 mM d-Glucose, pH 6.8). The diluted donor blood was centrifuged at 160 × g for 20 min at room temperature to obtain platelet rich plasma (PRP), which was fluorescently labeled with 5-Chloromethylfluorescein Diacetate (CMFDA, ThermoFischer C7025)(Baker et al. 1997; Sorensen et al. 2009; van der Wal et al. 2012; Wandall et al. 2008). Hereto, CMFDA dissolved in DMSO/PBS 1:5 (v/v) was added to the PRP at a final concentration of 100 µM and incubated for 1 h at room temperature (approximately 20 °C). Next, the fluorescent intensity of CMFDA per platelet was determined and platelets were transfused into recipient hamsters following cannulation of the superficial femoral artery under isoflurane anesthesia. Recipient hamsters were returned to their winter environment while remaining in darkness. Surgery-induced arousal and re-entry into torpor was confirmed with passive infrared sensors (Oklejewicz et al. 2001). Blood samples were collected and CMFDA labeled platelet amount was determined prior to and 10 min after transfusion and at euthanization one or more days after transfusion. At euthanization oral temperature and preceding activity pattern monitored with infrared sensors confirmed either torpor or arousal in hibernating animals and euthermia in the non-hibernating control animals.

### Flow cytometry analysis

Expression of P-selectin (CD62P) and CMFDA levels in platelets were analyzed by flow cytometry. One microliter of whole blood was diluted 1:25 (v/v) in phosphate buffered saline (PBS), and incubated with PE-labeled anti-CD62P (GeneTex 43,039) with or without 10 µM adenosine diphosphate (ADP) for 30 min in the dark. The activation was stopped by fixation with 2% formaldehyde in 300 µL PBS (v/v). Samples were acquired on a BD Biosciences Calibur flow cytometer equipped with CellQuest software (BD Biosciences). Platelet populations were gated on cell size using forward scatter (FSC) and side scatter (SSC). At least 50,000 platelets per sample were analyzed, or 180 s in case of low platelet counts (thrombocytopenia). Data was analyzed using Kaluza 1.2 software (Beckman Coulter). Not-activated samples were compared and activated samples were compared.

### Assessment of megakaryocyte rupture

Femurs were collected at euthanization and immediately fixated and decalcified in DECAL (containing < 15% formaldehyde, < 5% methanol, < 10% formic acid, Surgipath Leica microsystems) and stored at least 24 h at 4 °C. Bones were further decalcified [20% EDTA, 2% NaOH in PBS (*w*/*v*)] for 48 h, then sagitally cut in half, paraffin embedded and sectioned longitudinally. Sections of 4 µm were placed on poly-l-Lysine coated slides and incubated overnight at 60 °C. After further deparaffinization, bone marrow sections were stained with Hematoxylin–Eosin (HE) and embedded with Dibutylphthalate Polystyrene Xylene (DPX). Quantification of megakaryocytes was performed in 20 fields of view per femur section with light microscopy (Nikon Eclipse 50i) in a blinded fashion.

Plasma interleukin-1 alpha (IL-1α) concentration, as a marker of megakaryocyte rupture, was determined by ELISA according to the manufacturer's instructions (Hamster Interleukin 1A ELISA Kit, MBS006418 MyBiosource.com).

### Electron microscopy (Nanotomy)

Liver, spleen and lung were harvested upon euthanization and small blocks of approximately a cubed millimeter were immediately fixated in 2% glutaraldehyde plus 2% formaldehyde (v/v) in 0.1 M sodium cacodylate pH 7.3 for at least 24 h at 4 °C. After post-fixaton in 1% osmium tetroxide/1.5% potassium ferrocyanide in 0.1 M sodium cacodylate pH7.3 for 2 h at 4 °C, samples were dehydrated using ethanol and embedded in EPON epoxy resin. Sections of 60 nm were collected on formvar coated single slot grids and contrasted using 5% uranyl acetate in water for 20 min, followed by Reynolds lead citrate for 2 min. Next, scanning transmission electron microscopy (STEM) was performed on ~ 70,000 µm^2^ areas as described previously (Kuipers et al. 2016; Sokol et al. 2015) to generate a large field of view at high resolution, which is called ‘nanotomy’, for nano-anatomy. Data was acquired on a Supra 55 scanning EM (SEM; Zeiss, Oberkochen, Germany) using a STEM detector at 28 kV with 2.5 nm pixel size using an external scan generator ATLAS 5 (Fibics, Ottawa, Canada) as previously described (Kuipers et al. 2016; Sokol et al. 2015). After tile stitching, data were exported as an html file and uploaded to the online image database (www.nanotomy.org). Platelets were detected morphologically in fields of view of ~ 25 × 25 µm and confirmed by size and electron dense granular content on fields of view of ~ 8 × 8 µm. Representative images were processed similarly in opensource GIMP software (GNU Image Manipulation Program, The GIMP team, GIMP 2.8.10, www.gimp.org), as previously published for selecting areas of interest (Bijelic et al. 2017). In short, a mask was created over every platelet in one separate layer over the original image, colored red and set to opacity 75%. For quantification, platelets in liver sections were counted manually in a blinded fashion and divided by total section area size.

### Statistics and data presentation

Data are presented as mean ± SD. Statistical differences between groups were calculated using repeated measures ANOVA, one-way ANOVA and post-hoc Tukey analysis (Graphpad Prism v6, GraphPad Software) with *P* < 0.05 considered significantly different. Sum of squares F test was used to compare coefficients of non-linear regression curves. The same software was used to make the graphs.

## Results

### Platelets are stored during torpor and released upon arousal

**Fig. 1:**
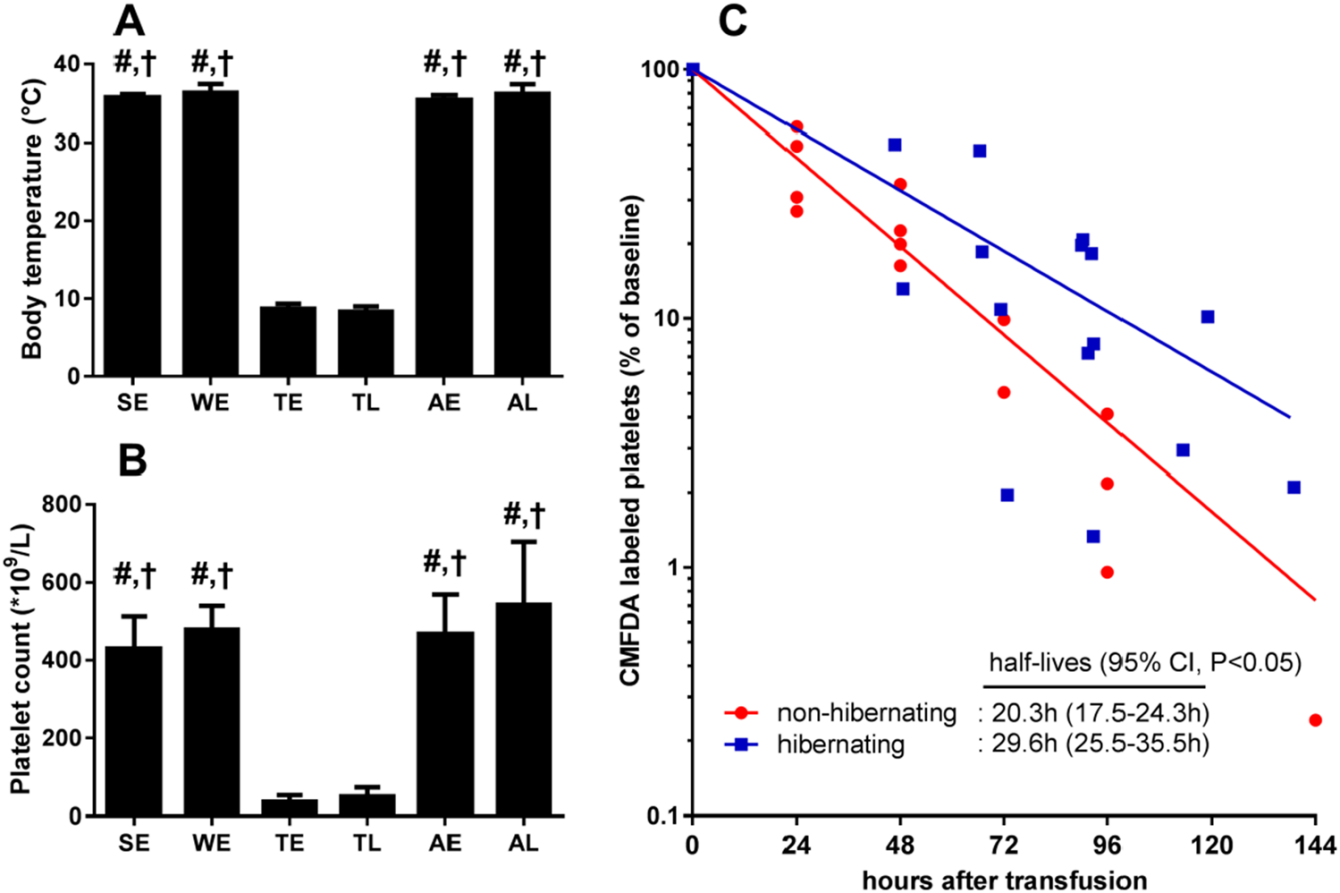
Reversible thrombocytopenia during torpor is via storage and release of platelets. **a** Oral body temperature at blood sampling confirmed torpor and arousal states of hibernating hamsters. **b** Platelet count reduces during torpor and rapidly recovers to euthermic level during 1.5 h of early arousal. **c** Decay of CMFDA labeled platelets expressed as % of baseline in non-hibernating hamsters and during torpor-arousal cycles in hibernating hamsters. Platelet survival is longer in hibernating (blue) than in non-hibernating hamsters (red) (*P* < 0.05). Sample sizes between *n* = 3 and *n* = 12, '#' and '†' denote difference from TE and TL, respectively (*P* < 0.05). *SE* summer euthermia, *WE* winter euthermia, *TE* early torpor (24–48 h), *TL* late torpor (> 48 h), *AE* early arousal (1.5 h), *AL* late arousal (> 8 h)

Summer and winter euthermic hamsters had body temperatures of 35.7 ± 0.4 °C and 36.3 ± 1.1 °C (Fig. 1a). During the torpor phase of hibernation, body temperature reduced to 8.2 ± 0.7 °C and recovered within 1.5 h of arousal to values not different from summer and winter euthermic values. Torpor was associated with a > 90% reduction in platelet count, reducing from 430 ± 82 in summer to 36 ± 17 × 10^9^/L early in torpor, which also recovered swiftly and fully upon arousal (Fig. 1b). Next, we determined whether platelet dynamics result from breakdown and de novo synthesis or from storage and release of platelets. Hereto, fluorescent CMFDA-labeled platelets were transfused in torpid hamster, which induces an arousal due to handling of the animal. Subsequently, hibernating animals re-entered torpor 38 ± 19 h following transfusion. Transfused non-hibernating winter euthermic hamsters served as controls. The number of circulating labeled platelets was assessed by flow cytometry 10 min after transfusion and at euthanization during torpor or arousal, at least 1 day after transfusion (Fig. 1c). Serial sampling demonstrated an exponential decay of labeled platelets with half-lives amounting 20.3 and 29.6 h in non-hibernating and hibernating hamsters (*P* < 0.05, Fig. 1c). Thus, labeled platelets of hibernating animals exit and return to the circulation similarly as non-labeled platelets, signifying that platelets were stored during torpor and subsequently released during arousal. Additionally, platelet survival is prolonged during hibernation.

### Rapid platelet recovery in arousal is not due to platelet synthesis or megakaryocyte rupture

**Fig. 2:**
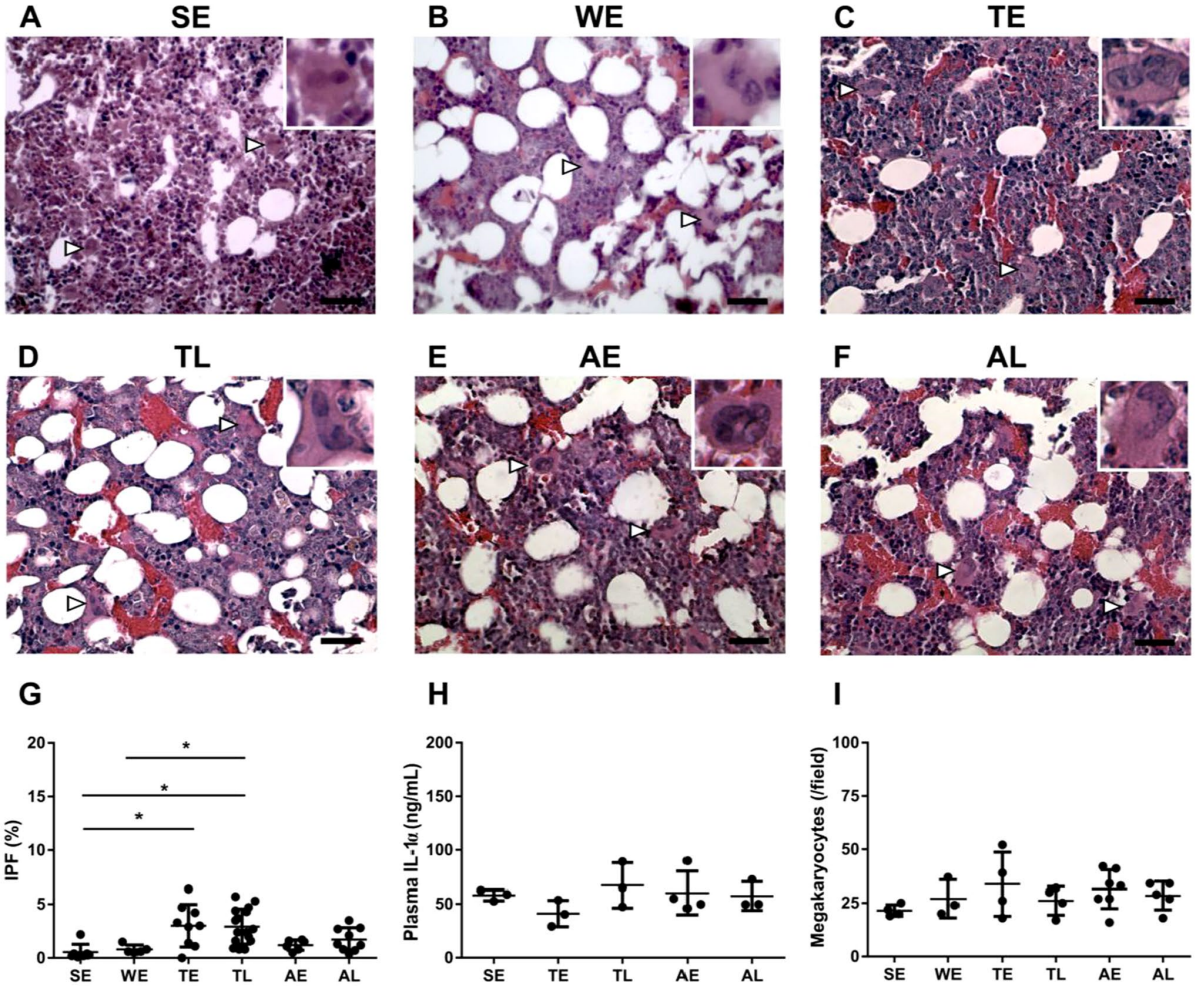
Absence of relevant de novo platelet synthesis in hibernating hamster. **a** Immature platelet fraction (IPF) as determined by flow cytometry, and **b** plasma interleukin 1α (IL-1α) ELISA measurements during different phases of hibernation. **c** Quantification of megakaryocyte numbers in sections of hamster femurs, expressed as average amount per 20 fields of view. **d–i** Representative fields of view of femur bone marrow from euthermic hamsters in summer or winter condition (SE, WE) or hibernating hamsters early or late in torpor and arousal (TE, TL, AE, AL). Two megakaryocytes are pointed out per image (arrowheads), one of them 2.5 × magnified in inset. HE staining, scale bars are 50 µm. Sample sizes between *n* = 3 and *n* = 16, *P < 0.05. *SE* summer euthermia, *WE* winter euthermia, *TE* early torpor (24–48 h), *TL* late torpor (> 48 h), *AE* early arousal (1.5 h), *AL* late arousal (> 8 h)

To further substantiate that platelet dynamics are governed by storage and release, rather than clearance and de novo synthesis of platelets, we determined the relative fraction of de novo synthetized platelets by measuring the immature platelet fraction (IPF) and the amount of megakaryocyte rupture, a mechanism by which IL-1α may rapidly produce platelets (Nishimura et al. 2015). IPF was low in euthermic animals, increased slightly in torpor and remained at this low number during arousal (Fig. 2a). IL-1α plasma levels (Fig. 2b) as well as bone marrow megakaryocyte numbers were similar in non-hibernating and hibernating hamsters that had undergone 9.3 ± 2.2 torpor bouts (Fig. 2c–i). Together, these results imply that de novo platelet synthesis, either by normal production or by megakaryocyte rupture, does not contribute to normalization of platelet amount during arousal.

### Platelet storage and release occurs in liver sinusoids, but not in spleen or lung

**Fig. 3:**
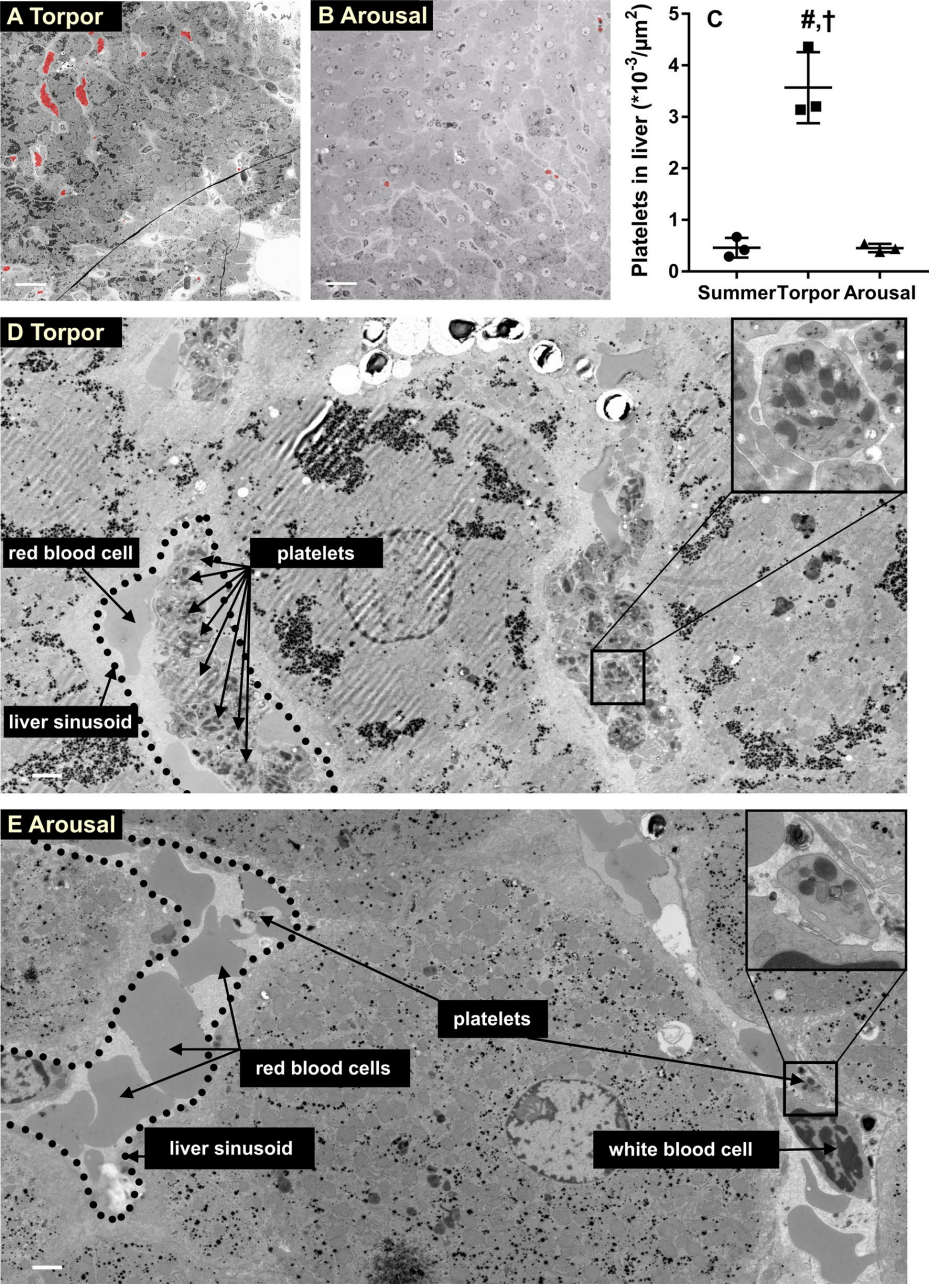
Platelets are stored in liver sinusoids during torpor and released during arousal. Representative images from large-scale scanning transmission electron microscopy (STEM) of hamster liver, according to the nanotomy protocol (full size dataset available online at nanotomy.org). **a** Low magnification of the entire section of liver from a hamster in torpor imaged by STEM demonstrating high density of platelets (overlay in red) in liver during torpor. Single and accumulated platelets are depicted in red overlay. **b** Low magnification of entire section of liver from a hamster in arousal demonstrating low density of platelets. **c** Increased platelet count per area of liver in electron microscopy sections in torpid hamsters compared to summer and arousal. **d** Liver sinusoids are filled with platelets during torpor. **e** During arousal, red blood cells are the predominant cell type in liver sinusoids with very few platelets present. Examples of different cell types are identified. Insets are zoomed on representative platelets. Scale bars represent 50 µm (**a**, **b**) and 5 µm (**d**, **e**), respectively. Sample sizes *n* = 3, '#' and '†' denote difference from summer and arousal, respectively (*P* < 0.05)

**Fig. 4:**
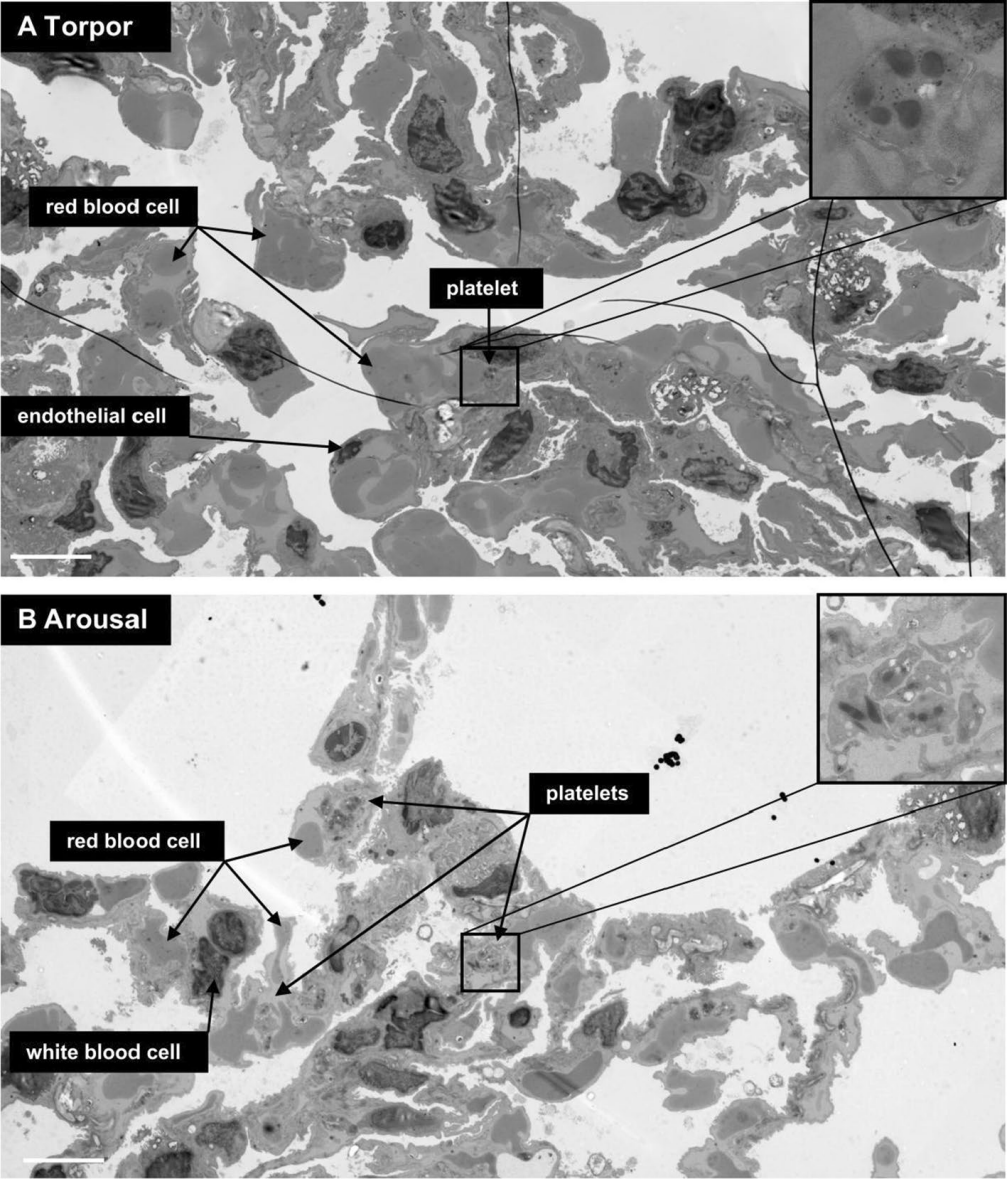
Platelet distribution in lung is similar in torpor and arousal. **a** Few platelets are seen in lung sections from torpid hamsters, whereas red blood cells are abundant. One platelet is pointed out within a capillary lumen (arrow). **b** Similarly, low amounts of platelets are present during arousal in capillaries of lung compared to abundant red blood cells. Examples of different cell types are identified. Insets are a zoomed on representative platelets. Scale bar is 5 µm. Full size dataset available online at nanotomy.org/OA/deVrij2020JCPB/

**Fig. 5:**
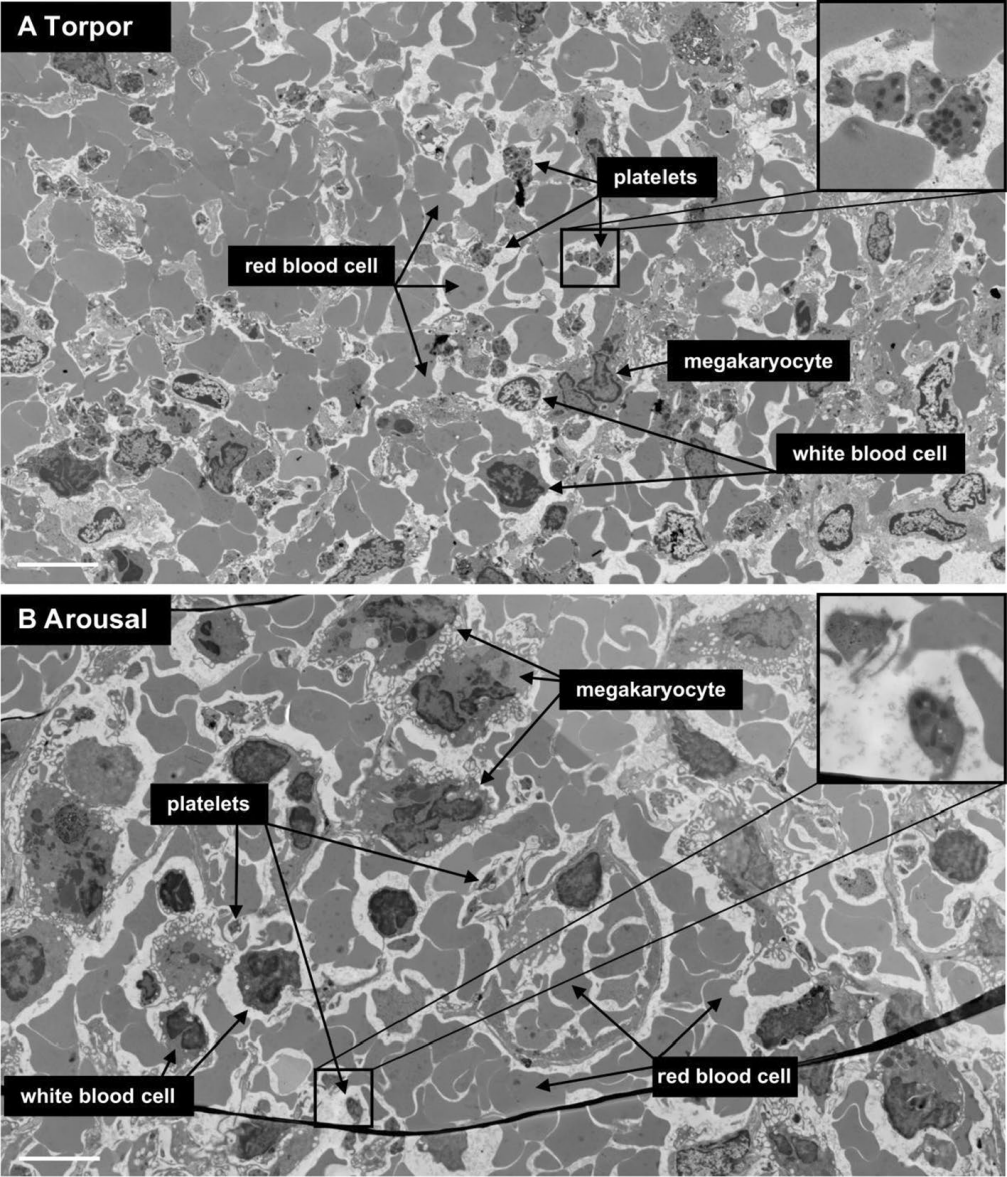
Similar platelet distribution in spleen during torpor and arousal. **a** Representative zoomed image of large-scale scanning transmission electron microscopy map of spleen from a torpid hamster. Red pulp from the spleen is in view with many red blood cells and dispersed platelets and several nucleated white blood cells. **b** Spleen from a hamster in arousal, with similarly high amount of red blood cells with dispersed platelets and white blood cells. Examples of different cell types are identified. Insets are a 3 × zoom on representative platelets. Scale bar is 5 µm. Full size dataset available online at nanotomy.org

Since platelets are stored during torpor and released in arousal, we next set out to determine their storage location. We used morphological identification by scanning transmission electron microscopy (STEM) and used Nanotomy (for nano-anatomy) (Kuipers et al. 2016; Sokol et al. 2015), which allows to create a single large-scale EM dataset that represents the equivalent of thousands of conventional transmission EM photos. Analysis of liver sections demonstrated a 7.9 fold increase in the number of platelets in torpor compared to arousal on the large-scale EM scan (Fig. 3a–c), while the number of platelets in aroused animals was similar to summer animals (Fig. 3c, Fig S1a). During torpor, platelets were localized primarily in liver sinusoids, often filling the entire sinusoid by forming platelet clusters and displacing erythrocytes (Fig. 3d). Conversely, in aroused animals, sinusoids were filled mainly with erythrocytes with the presence of an occasional, single platelet (Fig. 3e), similar to summer animals (Fig S1b). In addition, although rare, we also found platelets in the process of being phagocytosed by Kupffer cells in torpor and arousal and in the subendothelial space of Disse during torpor (Fig S2a, b). In contrast to liver, no changes were observed in lung and spleen. In lung, few platelets were found within capillaries, whereas red blood cells were abundantly present, which was similar for torpor and arousal hamster (Fig. 4a, b). Spleen red pulp contained a high amount of red blood cells with platelets distributed homogeneously in-between, both in torpor and arousal (Fig. 5a, b). Together, the number of platelets in liver increased strongly during torpor because of storage in sinusoids, which reversed rapidly during arousal, while numbers and distribution of platelets in lung and spleen were not affected by torpor or arousal.

### No signs of platelet activation or coagulation during torpor and arousal

**Fig. 6:**
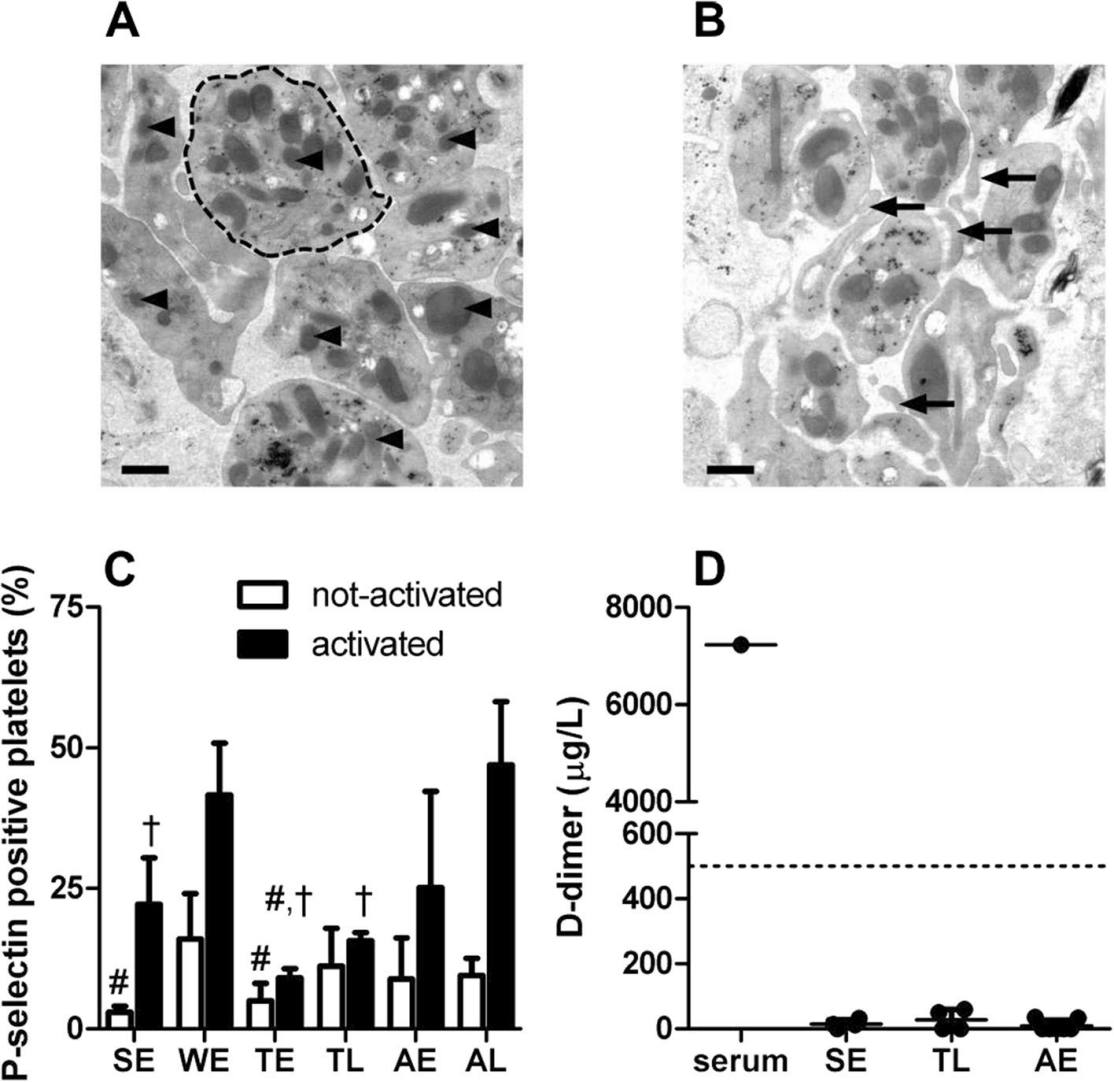
Thrombocytopenia in torpor is not linked to platelet activation or plasma coagulation activation. **a** Representative scanning transmission EM image of stored platelets in liver sinusoids during torpor with retained granules (arrowheads denote some example granules), platelets are not visibly degranulated. Dotted line encircles one platelet. **b** Stored platelets in sinusoids during torpor occasionally demonstrate extended membrane protrusions (filopodia, indicated by arrows). Scale bars denote 50 µm. **c** P-selectin expression, as a measure of platelet degranulation, of circulating platelets (“not activated”) and ADP-stimulated platelets (“activated”) expressed as % of total platelets. Activatibility of platelets is reversibly reduced in torpor. **d** Plasma D-dimer levels throughout hibernation. Hamster serum was used as positive control. Sample size *n* = 2–11, '#' denotes difference from WE, '†' denotes difference from AL, *P* < 0.05. *SE* summer euthermia, *WE* winter euthermia, *TE* early torpor (24–48 h), *TL* late torpor (> 48 h), *AE* early arousal (1.5 h), *AL* late arousal (> 8 h)

Large-scale EM analysis of torpid animals demonstrated that platelets stored in liver still contain granules (Fig. 6a), arguing against degranulation of platelets during torpor. However, we observed occasional membrane folds in platelets (Fig. 6b), mimicking filopodia, which may reflect platelet activation. To determine whether platelets and the coagulation system are activated during torpor, we determined platelet P-selectin expression on circulating platelets and plasma D-dimer levels (Fig. 6c, d). Circulating platelets in torpid and aroused hamsters had similar basal P-selectin expression, whereas activatibility gradually increased from torpor to early and late arousal, reaching levels similar to winter euthermia (Fig. 6c). D-dimer levels remained low in hibernating and non-hibernating animals and below threshold used in diagnosing thrombosis in humans (500 µg/L, Fig. 6d). Thus, thrombocytopenia during torpor is not associated with activation of platelets or the coagulation system. In addition, activatability of circulating platelets seems reversibly suppressed during torpor.

## Discussion

### Thrombocytopenia during torpor is governed by reversible storage and release in liver sinusoids

Here, we demonstrate unequivocally that thrombocytopenia during torpor is due to storage of platelets, followed by subsequent release of the same platelets upon arousal. Storage and release of platelets is principally evidenced by the observation that CMFDA labeled platelets injected prior to torpor, exit the circulation during torpor and recirculate upon arousal. To our knowledge, we are the first to demonstrate the platelet half-life in hamsters and its increase during hibernation. Half-life of transfused platelets in non-hibernating hamsters was 20.3 h. This is comparable to transfused platelets in non-hibernating squirrel and rat [approximately 29.5 h, and 20.9 h, respectively (Cooper et al. 2012)], but shorter than that in mouse [33.6 h (Olsson et al. 2005)] and human [107 h (Fritz et al. 1986)]. In addition, we show that platelets are mainly stored in liver sinusoids during torpor and released in arousal. The finding that storage and release governs platelet dynamics during hibernation is further supported by (1) absence of platelet activation or coagulation (i.e., no degranulation of platelets, low plasma D-dimer levels) and (2) no signs of de novo synthesis of platelets (i.e., low immature platelet fraction and low plasma IL-1α level and no signs of megakaryocyte rupture). The IPF level in hamster was between 0 and 6.4%, which is slightly wider than the range in mice and rat (0–1.6%, and 0.2–3%, respectively (de Vrij et al. 2014)) and similar to the IPF range in human and in ground squirrel (1.1–6.1% (Briggs et al. 2004) and approximately 0–7.7% (Cooper et al. 2012), respectively). Alterations in megakaryocyte ploidy might explain these minor changes in IPF (Mattia et al. 2002), but are less likely to contribute significantly to the fast and complete recovery of platelet count during arousal from its > 90% reduction in torpor. Although IL-1α is not specific for megakaryocyte rupture, and a rise could also be due to inflammation, as can arise secondary to infection, cell stress/tissue injury or ischemia or due to tumor development (Di Paolo and Shayakhmetov 2016), the absence of an increase in IL-1α makes megakaryocyte rupture very unlikely. Finally, we demonstrate that circulating platelets during hibernation are not activated, whereas platelets are suppressed in activatability during torpor, which reverses during arousal. Together, these results demonstrate that hibernators may shield themselves from thrombosis induced by immobility, low blood flow and low body temperature by reversibly suppressing the number and functionality of circulating platelets.

### Torpor induces platelet storage in liver with reduced activatibility of circulating platelets and absence of hemostatic activation

We demonstrate that liver sinusoids appear as the main compartment of platelet storage during torpor, from where platelets are released upon arousal. These findings match a recent study demonstrating increased amount of platelet glycoprotein Ib staining in liver of hibernating torpid ground squirrels, which reverses in arousal (Cooper et al. 2017). By large-scale electron microscopy (nanotomy) analysis (Kuipers et al. 2016; Sokol et al. 2015) we determined that the platelet storage location in torpor was not in lung or spleen, since the number of platelets did not change in these organs. In accord, we previously excluded a role of spleen in platelet storage by demonstrating that splenectomy before or during torpor is without an effect on platelet dynamics in hibernating hamster (de Vrij et al. 2014), which was recently corroborated in splenectomized squirrels (Cooper et al. 2017). Finally, our results exclude thrombosis and trapping of platelets within immune complexes or rosette cell formation as a contributor to platelet storage, since (micro)thrombi were absent in liver and lung, levels of D-dimer remained low throughout torpor and arousal, and platelets in torpid liver sinusoids were not degranulated and did not form large activated aggregates, but rather non-activated accumulations. Additionally, circulating platelets were not activated throughout hibernation, since circulating platelets of hibernating animals expressed similarly low levels of P-selectin. Moreover, the few platelets that circulated during torpor had a reduced activatability in response to ADP, as implied previously (de Vrij et al. 2014), which is in line with reduced aggregation of platelets from hibernating bears in response to ADP and other agonists (Arinell et al. 2017). Thus, the reversible platelet storage in liver sinusoids is due to platelet accumulation without evident signs of hemostatic activation.

### Reversible storage and release of platelets in liver sinusoids is likely mediated by margination

Margination depends on platelet-endothelium interaction and reflects a balance between adhesion and detachment. Several factors during torpor likely shift the balance to more adhesion. First of all, rheological forces stimulate margination because of substantial reductions in cardiac output and blood flow in torpor (Horwitz et al. 2013) and increase of hematocrit (Arinell et al. 2017), driving platelets to the vessel wall (Fitzgibbon et al. 2015; Reasor et al. 2012; Ruggeri 2009). Secondly, relative hypoxia during entrance in torpor (Carey et al. 2003) might lead to exocytosis of endothelial cell Weibel-Palade bodies, exposing P-selectin and releasing von Willebrand factor (Pinsky et al. 1996), thereby stimulating platelet adhesion to endothelial cells. Although local VWF level may hypothetically increase near the endothelium, systemic plasma level has been shown to decrease in ground squirrel during torpor (Cooper et al. 2016a). Thirdly, reduced temperature and blood flow may induce endothelium activation with increased expression of adhesion molecules (Awad et al. 2013; Li et al. 2014). In torpid hamster some endothelial activation markers increase (Talaei et al. 2012), whether this results from reduced flow and/or temperature is not yet known. One might hypothesize that platelets increase adhesiveness during torpor. Expression of adhesion markers has not been studied on stored platelets. However, the low P-selectin expression and suppressed activatability of circulating torpid platelets argues against relevant pro-adhesive effects of torpor on platelets themselves. Since platelets re-appear swiftly in circulation upon arousal, platelets likely detach from endothelium solely due to increases in blood flow and temperature. Hence, low blood flow, increased hematocrit and potentially increased adhesion molecule expression and local von Willebrand Factor levels due to relative hypoxia and low temperature, likely shift the balance towards platelet margination in torpor, which is rapidly reversed upon arousal.

### Implications

Here, we reveal margination of platelets to liver sinusoids as mechanism underlying the reversible storage and release of platelets in hibernation. We previously demonstrated that reversible thrombocytopenia in torpor depends on lowering of the body temperature (de Vrij et al. 2014). Lowering body temperature in non-hibernators also decreases cardiac output, blood flow and increases blood viscosity (Dudgeon et al. 1980; Van Poucke et al. 2014), favoring platelet margination (Reasor et al. 2012) and inducing a reversible thrombocytopenia (de Vrij et al. 2014). Hence, the effect of lowered body temperature on margination of platelets, which can lead to the drop in circulating platelet count, seems a widely conserved phenomenon that is not specific for hibernating species. Since accidental and therapeutic hypothermia in humans are also associated with thrombocytopenia (Jacobs et al. 2013; Mallet 2002; Mikhailidis and Barradas 1993; Morrell et al. 2008; Vella et al. 1988; Wang et al. 2015), knowledge of its underlying mechanism may aid in (hemostatic) management of hypothermia. Furthermore, the ability to pharmacologically induce reversible storage of platelets might be exploited for development of novel reversible antithrombotic strategies.

## Supplementary Information

Below is the link to the electronic supplementary material.

Fig S1 Platelet distribution in liver of hamsters in summer condition. Representative images from large-scale scanning transmission electron microscopy (STEM) of hamster liver in, according to nanotomy protocol (full size dataset available online at nanotomy.org). a Low magnification of the entire section of liver from a hamster in summer euthermia demonstrating low density of platelets (overlay in red). b Liver sinusoids are mainly filled with red blood cells in summer, occasionally platelets can be found. Insets are a zoomed on representative platelets. Scale bars are 20 µm (a) and 5 µm (b), respectively

Fig S2 Platelet phagocytosis in Kupffer cells and platelets in space of Disse during hibernation. a Electron microscopy imaging of hibernating hamster liver demonstrated some Kupffer cells, liver macrophages, in the process of phagocytosing platelets in torpor. Dashed line encircles a Kupffer cell. b In one instance we found a platelet in torpor in the space of Disse, the space between endothelial cells and hepatocytes, denoted by the space between dash-dotted lines. On the luminal side of the sinusoidal endothelium is a red blood cell. Insets are a zoomed on representative platelets, scale bars represent 1 µm

## Data Availability

Data used in figures available online at nanotomy.org.

## Abbreviations

SE: Summer euthermia
WE: Winter euthermia
TE: Early torpor
TL: Late torpor
AE: Early arousal
AL: Late arousal
CMFDA: 5-Chloromethylfluorescein diacetate
IL-1α: Interleukin-1 alpha
