# Supplementary figures and images for: Reversible thrombocytopenia during hibernation originates from storage and release of platelets in liver sinusoids
Source: J Comp Physiol B. 2021 Mar 4;191(3):603–15. doi: 10.1007/s00360-021-01351-3 (PMC8043940; doi:10.1007/s00360-021-01351-3)

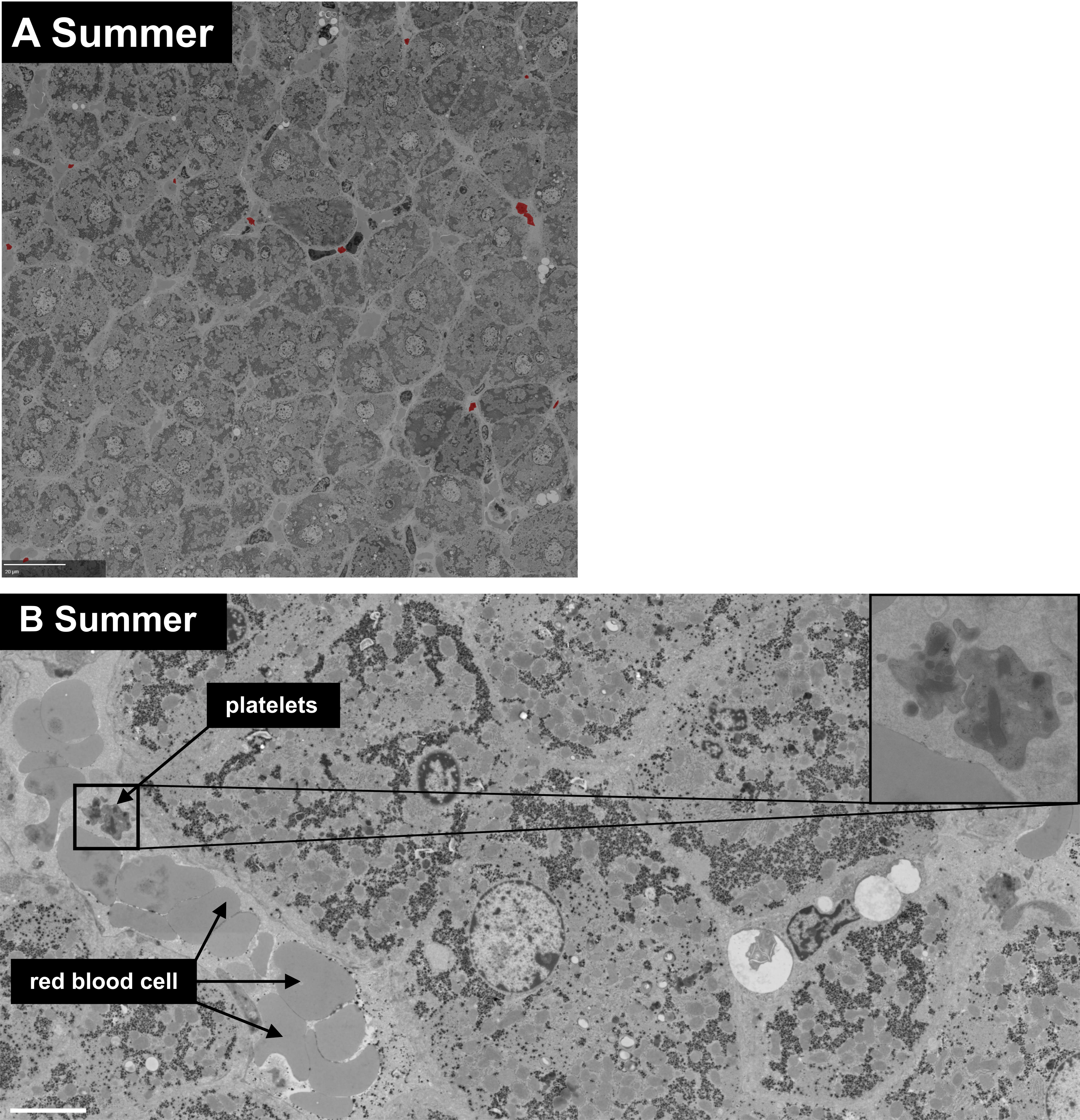

Supplement: Supplementary file 1 — Fig S1 Platelet distribution in liver of hamsters in summer condition. Representative images from large-scale scanning transmission electron microscopy (STEM) of hamster liver in, according to nanotomy protocol (full size dataset available online at nanotomy.org). a Low magnification of the entire section of liver from a hamster in summer euthermia demonstrating low density of platelets (overlay in red). b Liver sinusoids are mainly filled with red blood cells in summer, occasionally platelets can be found. Insets are a zoomed on representative platelets. Scale bars are 20 µm (a) and 5 µm (b), respectively [file 360_2021_1351_MOESM1_ESM.png]

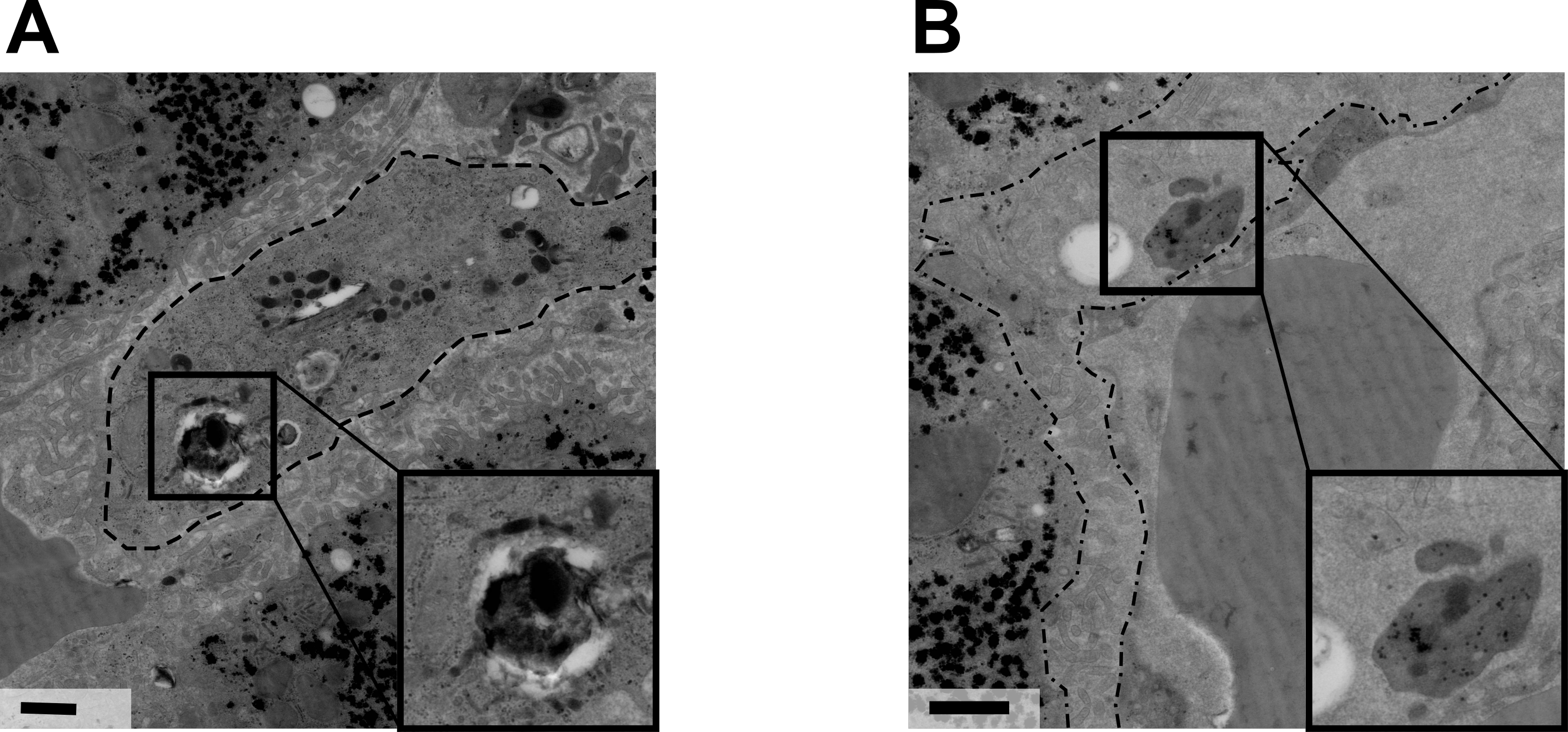

Supplement: Supplementary file 2 — Fig S2 Platelet phagocytosis in Kupffer cells and platelets in space of Disse during hibernation. a Electron microscopy imaging of hibernating hamster liver demonstrated some Kupffer cells, liver macrophages, in the process of phagocytosing platelets in torpor. Dashed line encircles a Kupffer cell. b In one instance we found a platelet in torpor in the space of Disse, the space between endothelial cells and hepatocytes, denoted by the space between dash-dotted lines. On the luminal side of the sinusoidal endothelium is a red blood cell. Insets are a zoomed on representative platelets, scale bars represent 1 µm [file 360_2021_1351_MOESM2_ESM.tif]
